# Pharmacokinetics and bioequivalence of *Withania somnifera* (Ashwagandha) extracts – A double blind, crossover study in healthy adults

**DOI:** 10.1016/j.heliyon.2023.e22843

**Published:** 2023-11-28

**Authors:** Se-Kwon Kim, Jayachandran Venkatesan, Priyank Rathi, Benny Antony

**Affiliations:** a College of Science & Technology, Hanyang University, ERICA Campus, Ansan, 11558, Republic of Korea; b Biomaterials Research Laboratory, Yenepoya Research Centre, Yenepoya (Deemed to be University), Mangalore, 575018, India; c Synergen Bio Private Limited, Sai Chambers, Shivajinagar, Pune, Maharashtra, 411003, India; d Arjuna Natural Pvt. Ltd., Innovation Centre, Behind ISRO, Erumathala P.O., Keezhmad, Kerala, 683 112, India

**Keywords:** Ashwagandha, *Withania somnifera*, Bioavailability, Bioequivalence, Withaferin A, Withanolide A, Withanoside IV, Total withanolides, AUC, T-half, Cmax, Pharmacokinetics

## Abstract

**Introduction:**

*Withania somnifera* (WS) or ashwagandha is an adaptogenic plant used extensively in traditional medicines and as a food supplement. Despite a long history of use and numerous clinical trials, the human pharmacokinetics of withanolides, the active phytochemicals in WS extracts, have not been fully evaluated. This study evaluated the oral pharmacokinetics and bioequivalence of active withanolides in human plasma after administration of a single dose of two commercial ashwagandha extracts containing equal amounts of total withanolides.

**Methods:**

This randomized, double-blind, single-dose crossover study of 16 healthy human volunteers evaluated the acute oral bioavailability of withanolides and the bioequivalence of two WS extracts, WS-35 and WS-2.5. WS-35 was standardized to total withanolides not less than 40% comprising not less than 35% withanolide glycosides and WS-2.5 was standardized to 2.5% withanolides. The clinical dosages were normalized to 185 mg of total withanolide in each extract at the bioequivalent dosages. The pharmacokinetic parameters of withanolide A, withanoside IV, withaferin A, and total withanolides were quantified in the blood plasma using a validated LC-MS/MS method.

**Results:**

The half-life, C-max, and mean residence time of the total withanolides were 5.18, 5.62 and 4.13 times significantly higher and had lower systemic clearance with WS-35 than with WS-2.5 extract. Considering the plasma AUC 0-inf of total withanolides per mg of each WS extract administered orally, WS-35 was 280.74 times more bioavailable than WS-2.5.

**Conclusion:**

The results of this study highlight the importance of withanolide glycosides in improving the pharmacokinetics of WS extracts. Owing to its superior pharmacokinetic profile, WS-35, with 35% withanolide glycosides, is a promising candidate for further studies on *Withania somnifera.*

**Clinical trial registration:**

CTRI/2020/10/028397 [registered on:13/10/2020] (Trial prospectively registered) http://ctri.nic.in/Clinicaltrials/pmaindet2.php?trialid=42149&EncHid=&userName=CTRI/2020/10/028397.

## 1 Introduction

*Withania somnifera* (L.) Dunal (WS), known as ‘ashwagandha’ in Ayurveda, is a perennial shrub belonging to the Solanaceae family. It is considered as one of the most important “Rasayana” herbs, with powerful adaptogenic and anti-stress properties. The herb is known to have a beneficial effect on the strength and function of the brain and nervous system while also enhancing sexual performance and reproductive function for a healthy life. As a powerful adaptogen, it enhances resilience to stress. In addition to its anti-stress and reproductive benefits, Ashwagandha is known to promote overall health and well-being. For instance, it is believed to have a positive impact on sleep, helping improve sleep quality and duration. Additionally, Ashwagandha has been found to have immunomodulatory properties, which means that it helps regulate the immune system and enhances its function. This makes ashwagandha a useful herb for supporting overall immune health and for combating illnesses [1,2].

WS contains various pharmacologically important constituents in its roots, leaves, fruit, and stems. Withanolides in WS, comprising steroidal lactones and withanosides, [3,4] are the active constituents responsible for several pharmacological activities [5]. Several withanolides, including withaferin A, withanolide A, withanoside IV, sitoindosides, and alkaloids have been identified and isolated from WS. These compounds are mainly localized in mature leaves and roots. The identified molecules included 62 major and minor primary and secondary metabolites from leaves and 48 metabolites from roots [6].

Ashwagandha extracts are in high demand because of their adaptogenic and immunomodulatory properties. The ability of ashwagandha to modulate the hypothalamus-pituitary axis and brain neurotransmitters has resulted in clinical findings related to reducing stress and anxiety, improving mood, and improving sleep quality. Withanolides also possess anti-inflammatory, immunomodulatory, anti-cancer, anti-diabetic, antioxidant, aphrodisiac, antibacterial, and anti-neurodegenerative properties. Among the various ashwagandha extracts available on the market, the standardization of actives, analytical methodologies, plant parts used, manufacturing methods, and recommended dosages vary considerably. These variables can potentially impact absorption characteristics and related therapeutic efficacy. Pharmacokinetic (PK) studies on the bioactive constituents of herbal drugs provide valuable information on their metabolism, dosage form, and directions for clinical use [7]. However, limited human data are available on the bioavailability and metabolism of withanolide.

This study evaluated the oral pharmacokinetics and bioequivalence of active withanolides in human plasma after administration of a single dose of two commercial ashwagandha extracts containing equal amounts of total withanolides. In the present study, the bioanalytical method used to determine withanolide levels in human blood plasma was validated according to the USFDA guidelines and was successfully applied to determine pharmacokinetic parameters in healthy volunteers. This study aimed to investigate the relationship between the ashwagandha extract and its oral bioavailability.

## 2 Methods

### 2.1 Study design

This bioavailability and bioequivalence single-center study employed a double-blind, balanced, randomized, single-dose crossover design. The study was conducted in healthy, adult human subjects under fasting conditionswith a minimum 7-day washout period between treatments to characterize the single-dose oral bioavailability and bioequivalence of two commercial ashwagandha extracts.

### 2.2 Investigational products

Two commercial *Withania somnifera* extracts were selected to study their oral bioavailability and bioequivalence at a dose of 185 mg total withanolides. The investigational materials used were WS-35 (standardized to 35% withanolide glycosides) and WS-2.5 (standardized to 2.5% withanolides).

Extract of dried roots and leaves of *Withania somnifera* WS-35 (Batch No. SH-PR 101 U/2003/S-16, Arjuna Natural Pvt. Ltd., India) was standardized to total withanolides NLT 40% comprising NLT 35% withanolide glycosides by HPLC. Trace metals were analyzed and heavy metals < 10 ppm, Lead < 0.5 ppm, Arsenic < 1 ppm, Cadmium < 1 ppm and Mercury < 1 ppm. Microbial assay was done and total platelet count < 1000 cfu/g, Yeast and Mould < 100 cfu/g, Salmonella Absent/25 g, *E. coli* Absent/10 g. *Withania somnifera* extract WS-2.5 (Batch No.: NBT/2001RM71, Natura Biotechnol, India) was standardized to withanolides NLT 2.5% by HPLC. Total heavy metals were NMT 10 ppm, Arsenic NMT 1 ppm, Cadmium NMT 1 ppm, Lead NMT 3 ppm and Mercury 0.1 ppm. Microbiological and pathogen analysis was done and Standard Plate count < 10000 cfu/gm, Yeast and Mould <100 cfu/gm and pathogens like *Escherichia coli*, Salmonella, *Pseudomonas aeruginosa* and *Staphylococcus aureus* were absent.

The choice of these two formulations was based on the highest strength enriched ashwagandha extract available and a low-strength ashwagandha with withanolide content. Dosage was formulated based on product specifications and 480 mg of WS-35 and 7400 mg of WS-2.5 provided an equal amount of 185 mg total withanolides. The investigational products were encapsulated in opaque green colored ‘0’ size capsules. All capsules had the same color, appearance, and packaging, and rice powder was used to achieve a consistent filling weight.

### 2.3 Ethical approval

The study protocol was approved by the Institutional Ethics Committee of the Sai Sneh Hospital and Diagnostic Center, Pune (DCGI Reg. No: ECR/989/Inst/MH/2017) before the commencement of the study. The study was conducted at Synergen Bio Private Limited, Pune, in November–December 2020 and the study participants were recruited from their database in accordance with the EC-approved protocol (No. 006-20, Date of Approval September 29, 2020) and clinical research guidelines established by the basic principles defined in the ICH-GCP guidelines (2016), the ICMR Ethical Guidelines for Biomedical Research on Human Subjects (2017), the Declaration of Helsinki (Fortaleza, Brazil, October 2013), G.S.R. 227(E) New Drugs and Clinical Trials Rules, 2019, and Guidelines for Bioavailability and Bioequivalence Studies, Central Drugs Standard Control Organization, March 2005. This clinical trial was registered in the Clinical Trial Registry of India (CTRI/2020/10/028397; registered on: 13/10/2020).

### 2.4 Participant screening and informed consent

Participants were screened within 21 days before dosing after obtaining their informed consent. The principal investigator, using English and/or Marathi as per the participants' preference, provided a detailed oral presentation on the study’s purpose, procedures, investigational products, potential risks, study requirements, and participant rights. Participants had the opportunity to ask questions and resolve any concerns. The interested participants signified their willingness to participate in the study by reading, signing and dating the consent document. These signed documents were securely filed in the study records and a copy was provided to the participant.

### 2.5 Selection of study participants

The participants, who were eligible when assessed against the inclusion and exclusion criteria for the study, were selected for the study.

#### 2.5.1 Inclusion criteria

Eligible participants included healthy male and non-pregnant, non-lactating female subjects aged between 18 and 45 years. Female subjects of childbearing potential were required to have recent negative serum beta human chorionic gonadotropin (β-HCG) and urine pregnancy tests, alongside the use of approved contraceptive methods. Acceptable forms of contraception included non-hormonal intrauterine devices, barrier methods with or without a spermicidal agent, surgical sterilization, or sexual abstinence. Male participants were required to agree to the use of appropriate contraceptive measures and refrain from sperm donation during the study period. Participants should have a body mass index (BMI) between 18.50 and 30.00 kg/m^2^, and a body mass not less than 50.00 kg. They should be in normal health based on personal medical history, clinical examination, and laboratory test results. Participants were also required to have a normal 12-lead electrocardiogram (ECG) recording, a normal chest X-Ray (P/A view), and a negative alcohol breath test result. Effective communication skills, written informed consent, adherence to the study’s protocol, provision of identity evidence, availability for the entire study duration, and the ability to fast for at least 14.00 h were also necessary for inclusion.

#### 2.5.2 Exclusion criteria

The exclusion criteria included participants with known hypersensitivity to ashwagandha or related drugs, individuals incapable of understanding the informed consent information, or those with a history or presence of significant cardiovascular, pulmonary, hepatic, renal, gastrointestinal, endocrine, immunological, dermatological, neurological, or psychiatric disease. Additionally, participants with a history or presence of alcoholism, drug abuse, or certain medical conditions such as asthma, gastric and/or duodenal ulceration, thyroid disease, adrenal dysfunction, organic intracranial lesion, or cancer were also excluded. Furthermore, individuals who had consumed certain substances such as tobacco, pan masala, gutkha, caffeine, or xanthine-containing foods within 48.00 h prior to the study were not included. Pregnant or nursing women, as well as those who had used implanted or injected hormonal contraceptives within 6 months prior to the study, were also excluded. Male volunteers unwilling to employ appropriate contraceptive measures or those willing to donate sperm during and up to 07 days after the completion of study were not included.

### 2.6 Sample size calculation

NCSS PASS was used for the sample size analysis process. The minimum number of participants needed for this study to detect a statistically significant effect size at a specific level of significance was calculated using a power analysis. Different factors like power (1-beta), significance level, and expected effect size were considered during the sample size analysis. An effect size with a 95% confidence level specified in the analysis, based on prior research served as the foundation for the sample size analysis. In order to confirm that the study would have sufficient sample power to detect significant relationships and differences among observations at little types I and II errors, sample size and power assessments were conducted. The minimum sample size needed to have a power of 90% at a significance level of 5% with an effect size 0.6 is 12. With an estimated drop out of 20%, a total number of 16 subjects was required for the study.

### 2.7 Randomization, blinding and dispensing of investigational products

A total of 16 individuals were selected for this study, each assigned a unique number from 01 to 16. Group assignments during the crossover period were determined using a randomization schedule generated by a biostatistician using the PROC PLAN procedure of SAS (SAS Institute Inc., U.S.A.) version 9. The investigational products were dispensed by the pharmacy custodian in coded bottles, following the randomization schedule while maintaining blinding. Dispensing records were securely stored in the pharmacy with controlled access throughout the study. Before dosing, a randomization code break envelope was prepared by the pharmacist, indicating the subject number, period number, and protocol number. This study was conducted as a double-blind trial, ensuring that both subjects and investigators remained unaware of the treatment received to prevent bias. The test and reference formulation closely resembled each other in color and appearance, facilitating blinding.

### 2.8 Study procedure

Subjects were accommodated in the clinical facility for a minimum of 11 h prior to dosing to ensure a fasting period of at least 10 h before the administration of the investigational product and for pre-dose blood sample collection. They remained in the facility post-dose until at least 24 h in each period. Following the administration of each product, there was a washout period of 7 days (equivalent to at least five elimination half-lives) to minimize the presence of measurable drug levels before dosing in the subsequent period. All subjects were instructed to avoid caffeine and/or xanthine-containing foods or beverages (e.g., coffee, tea, chocolate, caffeine-containing sodas) and grapefruit and/or its juice, as well as poppy-containing foods, for at least 48 h prior to check-in for each period and throughout their stay in the facility. Subjects received a standardized meal on the check-in day and the meal content and quantity were consistent for all periods. Drinking water was prohibited from 1 h before dosing until 1 h after dosing (except for approximately 240 mL of water provided during dosing) in each period.

### 2.9 Collection and processing of plasma samples

After an overnight fasting for a minimum of 10 h, WS-35 or WS-2.5 was orally administered according to the randomization schedule in each period. Blood samples were collected at various time points: pre-dose (0 h), as well as at 0.25, 0.5, 0.75, 1, 1.5, 2, 2.5, 3, 3.5, 4, 5, 6, 9, 12, 16, and 24 h post-dosing to measure the plasma concentrations of ashwagandha extract. Each period involved the collection of a total of 17 blood samples per subject. Blood samples were obtained through an indwelling cannula placed in a forearm vein using disposable syringes. An intravenous indwelling cannula was kept *in situ* as long as possible during the 24-h in-house stay in each period. At each sampling time point, 5 mL of blood was collected into pre-labeled sample collection tubes containing lithium Heparin anticoagulant. Centrifugation of the blood samples, under refrigeration, commenced within 1 h of collection. After centrifugation, the separated plasma was transferred to pre-labeled polypropylene tubes, with each tube indicating project number, subject number, period number and sampling time point. These polypropylene tubes were stored at −80 °C ± 10 °C within 1 h of the centrifugation process. The blinded plasma samples were later analyzed for actives of ashwagandha using a high-performance liquid chromatography (LC-MS-MS) method.

### 2.10 Bioanalytical procedures

#### 2.10.1 Chemicals and reagents

Reference standards for withanoside IV (USP grade, Lot no:1719532), withaferin A (analytical standard, Lot no:89910), and withanolide A (USP grade, Lot no:1719500) were purchased from Sigma-Aldrich (St. Louis, MO, USA). The internal standard, Tianeptine Cas No. 66981-73-5, was procured from Sigma-Aldrich (St. Louis, MO, USA). Methanol, Acetonitrile, *o*-phosphoric acid, and formic acid (LCMS grade) were purchased from Merck, India and water was collected from a Milli-Q organic-free water system (Millipore Corporation, Bedford, MA, USA). The OasisR HLB solid-phase extraction cartridge was obtained from Waters (Milford, MA).

#### 2.10.2 Instrumentation

A Triple quadrupole mass spectrometer coupled with an Acquity H-class UPLC with a PDA detector (Waters Corporation, Milford, U.S.A) was used to analyze withanoside IV, withaferin A and withanolide A in the plasma samples. Tianeptine was used as the internal standard [8]. Acquity UPLC BEH phenyl C18 column 100 × 2.1 mm L.D., 1.7 μm, (Waters, Milford, MA, USA) was used. The gradient mobile phase was 0.1% formic acid and 0.1% formic acid in acetonitrile. The gradient program was 5% B, 7.20 min, 45% B, 10–11.2 min, 80% B, 12–16 min, 5% B with flow rate 0.300 mL/min. Mass spectrometry analysis was performed in the positive and negative ESI multiple reaction monitoring (MRM) scan type with negative ion mode for withanoside IV and positive ion mode for withaferin A, withanolide A internal standard. The mass spectrometer parameters are listed in Table 1.

**Table 1:** Mass spectrometer parameters.

Parameters	Withanoside IV	Withaferin A	Withanolide A	Tianeptine
Molecular weight (Da)	782.9	470.6	470.6	458.933
Parent mass (*m*/*z*)	827.4408	471.1711	471.185	437.0904
Product mass (*m*/*z*)	763.3592	94.95	263.1045	292.0991
Collision energy (eV)	32	50	26	38
Cone voltage (eV)	30	22	24	22

#### 2.10.3 Preparation and extraction of standards and samples

Standard stock solutions of withanoside IV (1 mg/mL, w/v), withaferin A (1 mg/mL, w/v), withanolide A (1 mg/ml, w/v), and tianeptine (internal standard) (1 mg/mL, w/v) were prepared in methanol. The 50 μL of working solutions of standards were added to 450 μL of blank plasma to obtain withanoside IV and withanolide A concentration levels of 1.00–640 ng/mL and withaferin A concentration levels of 0.50–320 ng/mL. The sample preparation involved solid-phase extraction with OasisR HLB 1cc cartridges. Calibration standards and QC samples were spiked with 25 μL internal standard (2.00 μg/mL) and diluted with 500 μL of 4% ortho phosphoric acid in Eppendorf tubes, and samples were loaded into the preconditioned SPE cartridge. The cartridges were then washed with water (1 ml of water). Withanoside IV, withaferin A and withanolide A eluted with 3 × 1 ml of methanol. The solvent was then evaporated using a high-performance personal evaporation system (Genevac). The residue was reconstituted with 0.5 ml of acetonitrile: water containing 0.1% formic acid (1:1) and filtrate was injected into the LC-MS/MS system.

#### 2.10.4 Method validation

**Fig. 1:**
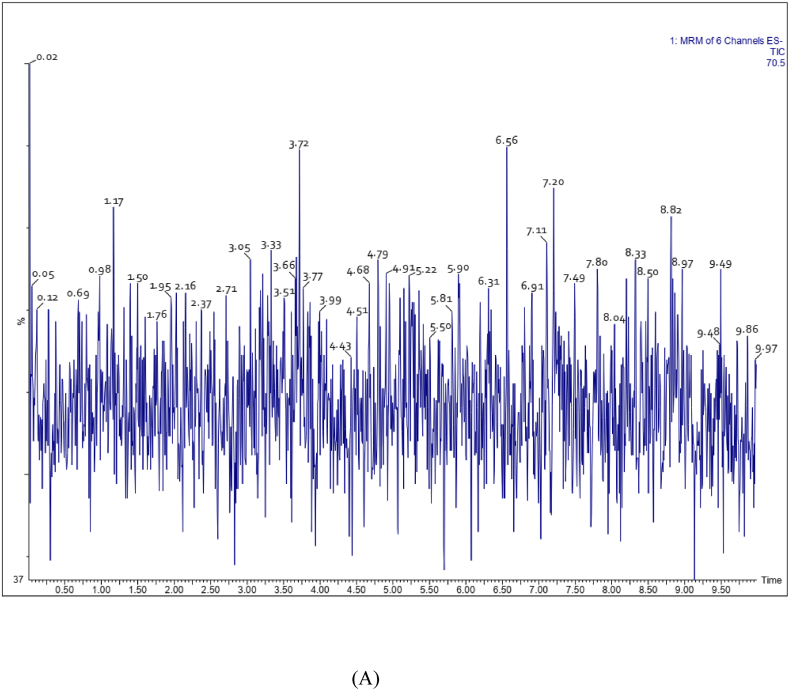

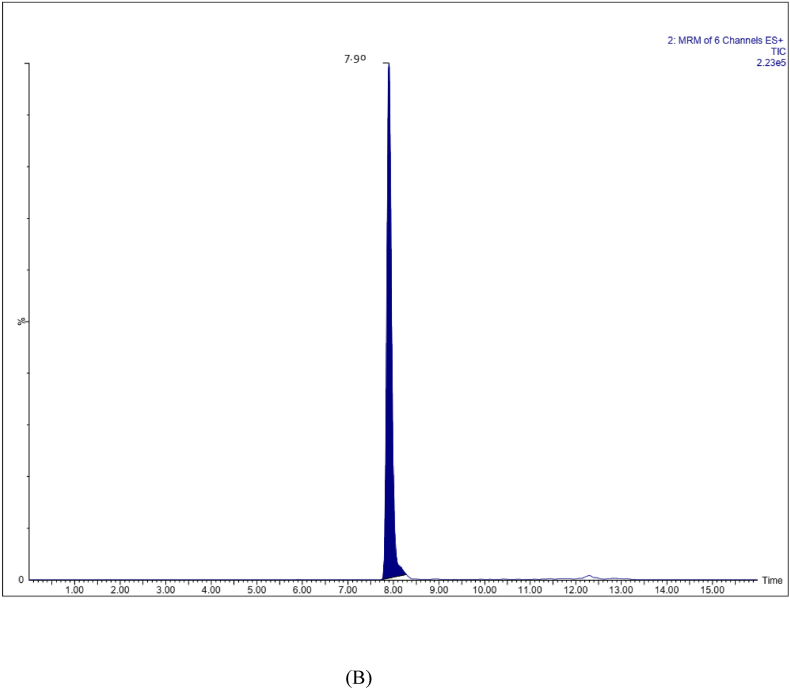

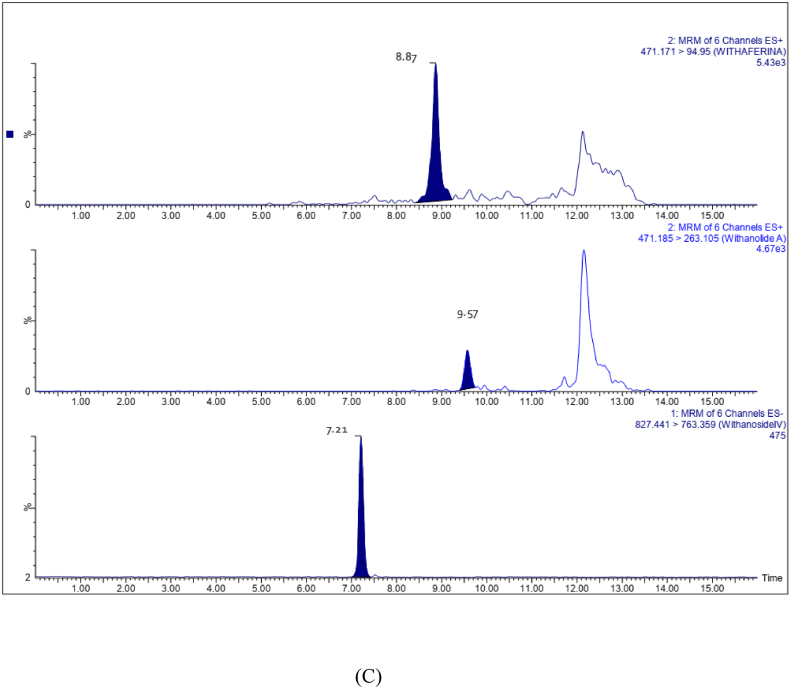
Representative UPLC -ESI-MRM chromatograms of extracted blank human plasma (A) chromatogram of blank human plasma without internal standard (B) chromatogram of blank human plasma spiked with internal standard (C) chromatogram of blank human plasma spiked with withaferin A, withanolide A and withanoside IV at lower limit of quantitation (LLOQ) level.

The method was validated in accordance with the industrial guidelines for bioanalytical method validation of the US Food and FDA [9]. Drug-free human plasma containing K2 EDTA anticoagulant was obtained from the blood bank and stored frozen at −80 ± 10 °C until analysis. The retention times of analytes in the interfering substances were absent from chromatograms obtained from six different sources of blank plasma spiked with target analytes at the lower limit of quantitation (LLOQ) levels. Representative chromatograms are shown in Fig. 1(A–C). The calibration curve regressions for withanoside IV, withanolide A and withaferin A exhibit strong linearity with coefficients of determination (r2) of 0.9990, 0.9969, and 0.9985 respectively. The LLOQ was calculated by the analyte response at the LLOQ ≥ five times the analyte response of the zero calibrator. The LLOQ of withanoside IV and withanolide A was 1.0 ng/ml and that of withaferin A 0.5 ng/ml in plasma. The LOD for withanoside IV, withaferinA and withanolide A is 0.5, 0.5 & 0.1 ng/ml. Intra/inter day precision and accuracy determined using QC samples at four concentrations with six replications spiked in the blank plasma were found to be within acceptable range (±20% & 80–120% for LLOQ, <15% & 85–115% for other QC’s of nominal concentration) (Table 2). The Intra batch precision (coefficient of variation) ranged from 6.95 to 17.98% for withanoside IV, 7.27–16.62% for withaferin A and 3.47–15.85% for withanolide A respectively. The accuracy (%) ranged from 89.18 to 96.63% for withanoside IV, 92.99–102.97% for withaferin A and 94.09–107.57% for withanolide A respectively. The Inter batch coefficients of variation ranged from 1.61 to 10.76% for withanoside IV, 0.06–6.77% for withaferin A and 1.46–5.30% for withanolide A. The accuracy ranged from 95.33 to 100.57% for withanoside IV, 93.05–100.21% for Withaferin A and 92.81–105.12% for withanolide A respectively. The percentage extraction recoveries ranged from 93.06 to 93.60% for withanoside IV, 91.72–93.54% for withaferin A and 91.23–91.61% for withanolide A respectively (Table 3). The percentage of matrix effects ranged from 91.00 to 94.00% for withanoside IV, 90.80–94.04% for withaferin A and 90.88–93.42% for Withanolide A respectively (Table 2, Table 3). Since the blood samples were collected in K2 EDTA tubes, the impact of anticoagulants on quantification was also analyzed and found no interfere with the assay performance. The stability of LQC, MQC & HQC with six replicate samples of withanoside IV, withaferin A and withanolide A were assessed under conditions such as long term stability (−80 °C for 60 days), three freeze (−20 °C) thaw cycles (at room temperature), short-term room temperature storage and autosampler stability (Table 4). In all stability tests, the samples exhibited a recovery rate of 90–105%. These results confirm the stability of withanoside IV, withaferin A, and withanolide A under the specified conditions.

**Table 2:** Precision and accuracy data for withanoside IV, withanolide A and withaferin A.

Analytes	Nominal concentration (ng/mL)	Intra-day (n = 6)			Inter- day (n = 18)		
		Observed concentration (ng/ml)	Precision	Accuracy	Observed concentration (ng/ml)	Precision	Accuracy
		Mean ± SD	(% CV)	Mean ± SD	Mean ± SD	(% CV)	Mean ± SD
Withanoside IV	LLOQ (1)	0.88 ± 0.14	16.29	87.80 ± 16.29	0.96 ± 0.08	8.33	96.11 ± 8.29
	LQC (3)	3.01 ± 0.34	11.41	100.12 ± 11.42	2.91 ± 0.09	3.00	96.86 ± 2.83
	MQC (30)	28.75 ± 3.41	11.87	95.84 ± 11.38	29.50 ± 0.80	2.71	98.33 ± 2.65
	HQC (75)	67.95 ± 3.85	6.94	90.60 ± 5.14	74.33 ± 5.91	7.95	99.01 ± 7.75
Withanolide A	LLOQ (1)	0.95 ± 0.16	17.18	94.66 ± 16.23	0.90 ± 0.05	4.99	90.43 ± 4.08
	LQC (3)	3.17 ± 0.10	3.24	105.73 ± 3.37	3.00 ± 0.16	5.24	100.03 ± 5.23
	MQC (30)	32.22 ± 1.15	3.57	107.43 ± 3.81	31.51 ± 1.00	3.18	105.03 ± 3.35
	HQC (75)	78.99 ± 2.78	3.52	105.32 ± 3.70	79.32 ± 0.38	0.48	104.76 ± 1.69
Withaferin A	LLOQ (0.5)	0.50 ± 0.08	16.39	99.53 ± 16.25	0.52 ± 0.04	7.19	104.57 ± 7.24
	LQC (1.5)	1.44 ± 0.12	8.07	95.73 ± 7.70	1.47 ± 0.07	5.03	97.74 ± 4.76
	MQC (15)	14.35 ± 1.00	6.98	95.65 ± 6.67	14.48 ± 0.20	1.36	96.55 ± 1.31
	HQC (37.5)	38.02 ± 2.93	7.71	101.38 ± 7.82	37.39 ± 2.51	6.72	98.33 ± 5.16

LLOQ- Lower limit of quantification.

LQC- Lower quality control.

MQC-Middle quality control.

HQC- Higher quality control.

**Table 3:** Extraction recovery and matrix effect of withanoside IV, withanolide A and withaferin A.

Analytes	Nominal concentration (ng/mL)	R1	R2	R3	Recovery (%)	Matrix effect (%)
		Mean ± SD (n = 3)	Mean ± SD (n = 3)	Mean ± SD (n = 3)	Mean ± SD (n = 3)	Mean ± SD (n = 3)
Withanoside IV	LQC (3)	14.67 ± 0.58	15.67 ± 0.58	16.67 ± 0.58	93.61 ± 0.24	94.00 ± 0.21
	MQC (30)	170.00 ± 5.29	182.00 ± 3.00	200.00 ± 2.00	93.39 ± 1.52	91.00 ± 1.31
	HQC (75)	473.33 ± 5.03	508.67 ± 6.11	544.00 ± 7.21	93.06 ± 0.77	93.51 ± 1.14
Withanolide A	LQC (3)	97.00 ± 1.00	106.33 ± 1.53	117.00 ± 1.00	91.23 ± 0.42	90.88 ± 0.56
	MQC (30)	1308.33 ± 9.29	1433.67 ± 9.29	1534.67 ± 5.51	91.26 ± 1.02	93.42 ± 0.37
	HQC (75)	3371.33 ± 8.14	3680.00 ± 8.54	3993.33 ± 8.33	91.61 ± 0.27	92.15 ± 0.17
Withaferin A	LQC (1.5)	275.33 ± 5.51	294.33 ± 4.04	313.00 ± 4.36	93.54 ± 0.66	94.04 ± 0.71
	MQC (15)	1825.00 ± 2.00	1989.67 ± 7.37	2191.33 ± 5.86	91.72 ± 0.27	90.80 ± 0.55
	HQC (37.5)	4382.67 ± 5.86	4777.33 ± 8.62	5229.33 ± 6.81	91.74 ± 0.05	91.36 ± 0.13

R1 represents analytes spiked before extraction.

R2 represents analytes spiked after extraction.

R3 represents analytes prepared in injection solvent.

Recovery (%) was calculated as R1/R2 × 100.

Matrix effect (%) was calculated as R2/R3 × 100.

**Table 4:** Stability data of Withanoside IV, Withanolide A & Withaferin A

Analytes	Nominal concentration (ng/mL)	Long term stability (%±SD)	Freeze thaw stability (%±SD)	Short term stability (%±SD)	Autosampler stability (%±SD)
WithanosideIV	LQC (3)	90.11 ± 3.66	92.39 ± 4.68	90.06 ± 3.06	106.67 ± 7.60
	MQC(30)	103.05 ± 7.29	91.39 ± 5.36	98.97 ± 9.90	104.10 ± 8.38
	HQC (75)	95.28 ± 6.77	96.73 ± 8.88	98.04 ± 9.17	95.25 ± 5.76
WithanolideA	LQC (3)	102.17 ± 8.43	96.67 ± 8.30	96.33 ± 8.34	97.22 ± 8.80
	MQC(30)	99.72 ± 8.04	96.21 ± 7.39	96.78 ± 8.19	104.77 ± 9.89
	HQC (75)	97.63 ± 8.69	97.27 ± 8.72	97.26 ± 8.61	96.10 ± 7.07
WithaferinA	LQC (1.5)	103.56 ± 7.85	95.56 ± 8.81	97.11 ± 9.92	98.44 ± 4.23
	MQC(15)	104.51 ± 8.48	103.44 ± 8.49	105.06 ± 9.31	103.12 ± 6.57
	HQC (37.5)	95.28 ± 8.58	94.78 ± 8.87	94.20 ± 8.35	94.61 ± 7.49

Data are expressed as means % ± SD (n = 6), Observed con/nominal con *100.

LQC- Lower quality control.

MQC-Middle quality control.

HQC- Higher quality control.

### 2.11 Pharmacokinetic analysis

Pharmacokinetic end point and the pk variables were calculated using the ‘ncappc’ package in R [9]. The pharmacokinetic parameters Cmax, AUC0-t and AUC0-∞ of actives of Ashwagandha Extracts were compared after log transformation. Other pharmacokinetic parameters, Tmax, t1/2, Kel and extrapolated AUC of actives of ashwagandha extracts were obtained and reported.

### 2.12 Safety assessment

Safety laboratory assessments were performed during the participant screening and at the end of the study. Adverse events were recorded by spontaneous reporting and non-directive questioning of each participant during each visit.Physical examination, vital signs, laboratory investigations, and ECG were performed during the clinical laboratory evaluation.

### 2.13 Statistical evaluation

The present study was a single-dose study that included the calculation of the area under the curve to the last quantifiable concentration (AUC_0-*t*_ and AUC_0-∞_), T_max_, and C_max_. Additionally, the elimination rate constant (K_e_), elimination half-life (*t*_1/2_), and other parameters were estimated. Bioequivalence is generally determined by comparing the population averages of a bioequivalence metric, such as AUC and C_max_. This approach, termed *average bioequivalence,* involves the calculation of a 90% confidence interval for the ratio of averages (population geometric means) of the bioequivalence metrics for the test and reference drug products [10]. Mean ratios and 90% confidence intervals were calculated for PK variables.

A 2 × 2 crossover design using *t*-test was conducted on the data using NCSS 2021 to determine the bioequivalence as well as to estimate the variability in the average response of the treatment and reference to the pharmacokinetic parameters at a significant level of 0.05. A 2 × 2 crossover design using non-parametric Mann-Whitney *U* Test was used to determine the variability in Tmax between the treatments. The Fieller’s confidence interval test was used to check the bioequivalence of the pharmacokinetic parameters, except for Tmax. The confidence interval approach, originally proposed by Westlake (1981) [11], suggests that bioequivalence can be determined if a (1 − 2***α***) × 100% confidence interval for the difference ***μμ****TT* − ***μμ****RR* or ratio ***μμ****TT*/***μμ****RR* falls within predefined acceptance limits. The Tmax was estimated as the sampling time point at which Cmax occurred. Its distribution, either on the original or the log scale, rarely follows a normal distribution. As a result, both the EMEA guidance (2001) and the WHO draft guidelines (2005) recommend using a 90% non-parametric confidence interval for assessment of average bioequivalence based on Tmax [12]. Non-parametric Schuirmann’s Wilcoxon-Mann-Whitney Equivalence Test using Two One-Sided Tests (TOST) was performed to determine the Tmax. A paired *t*-test analysis was conducted on safety test parameters (haemoglobin, RBC, PCV, WBC count, neutrophils, lymphocytes, eosinophils, monocytes, basophils, platelet count, SGOT, SGPT, alkaline phosphatase, total bilirubin, blood urea and creatinine) to understand any significant variability between the observations during screening and post-test period.

## 3 Results

**Fig. 2:**
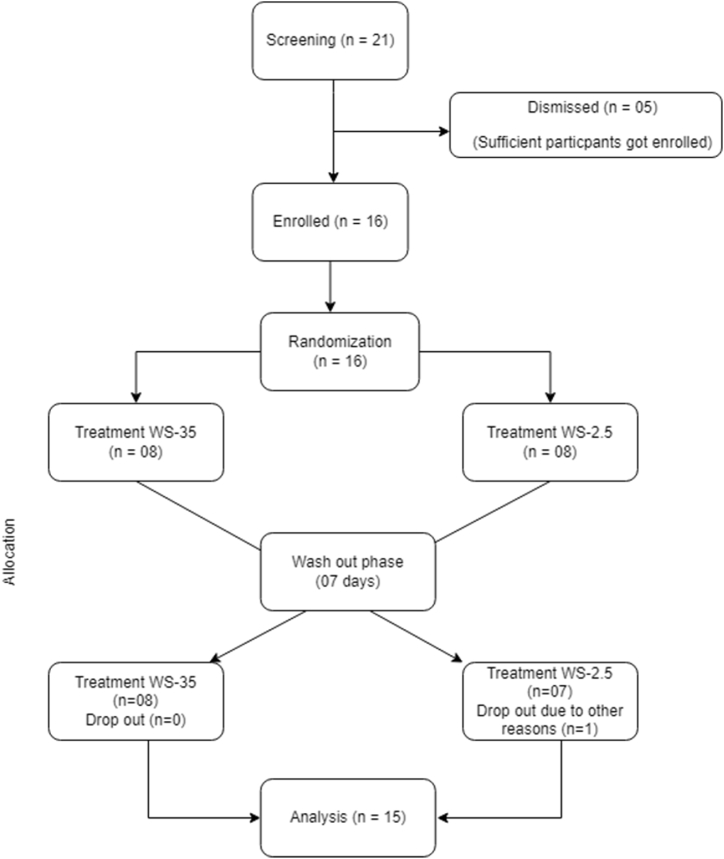
Study flow diagram.

Sixteen healthy participants were enrolled in the study to determine the pharmacokinetic profile of the ashwagandha extract. One subject dropped out during the second visit, 15 subjects completed the crossover study. The data of 15 subjects who completed the cross over study were analyzed. No participant data was removed due to outliers. The study flow diagram is represented in Fig. 2. The mean age, height, weight and BMI of the participants in the study were 33.8 years, 168.9 cm, 67.38 kg and 23.48 kg/m^2^ respectively. All subjects enrolled were males and of Asian origin. All subjects were non-smokers, non-vegetarians and non-alcoholic.

**Fig. 3:**
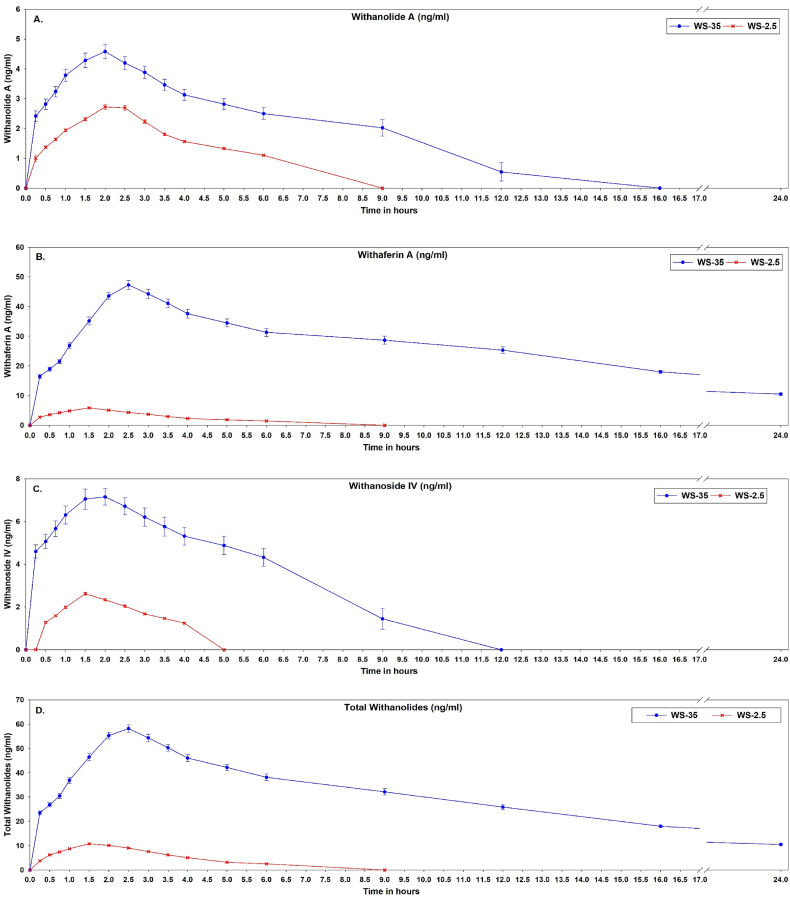
Plasma concentration –time curve (A) Withanolide A. (B) Withaferin A. (C) Withanoside IV. (D) Total withanolides after administration of formulations of *Withania somnifera* extracts WS-35 and WS-2.5.

Plasma concentrations of withanolide A, withaferin A, withanoside IV and total withanolides at various time intervals following a single oral dose of 185 mg of total withanolides from WS-35 and WS-2.5 extracts in healthy subjects are shown in Fig. 3(A–D). The concentration of total withanolides in the blood plasma was calculated as the sum of the withanolide A, withaferin A and withanoside IV concentrations. The pharmacokinetic parameters of withanolide A, withaferin A, withanoside IV, and total withanolides in blood plasma with WS-35 and WS-2.5. are represented in Table 5, Table 6, Table 7, Table 8.

**Table 5:** Comparison of pharmacokinetic parameters of withanolide A concentration in blood plasma with *Withania somnifera* extracts.

Parameters	WS-35	WS-2.5	LSM Difference ± SE	95% CI		P value
	LSM ± SE	LSM ± E		Lower	Upper	
AUC 0-inf∗	69.226 ± 15.42	17.11 ± 0.573	52.116 ± 15.34	18.975	85.256	0.00477
AUC 0-t∗	28.601 ± 2.688	10.661 ± 0.154	17.94 ± 2.681	12.147	23.732	0.00001
AUC ext∗	40.625 ± 13.149	6.449 ± 0.504	34.176 ± 13.095	5.886	62.466	0.02160
Clearance∗	3.809 ± 0.486	10.963 ± 0.356	−7.154 ± 0.611	−8.475	−5.834	<0.001
Cmax∗	4.736 ± 0.219	2.928 ± 0.061	1.808 ± 0.245	1.279	2.337	0.00001
Ke∗	0.094 ± 0.016	0.182 ± 0.012	−0.088 ± 0.021	−0.133	−0.042	0.00107
Lambda Z∗	0.094 ± 0.016	0.182 ± 0.012	−0.088 ± 0.021	−0.133	−0.042	0.00107
Mean Residence Time∗	15.98 ± 3.053	6.238 ± 0.299	9.742 ± 3.044	3.167	16.318	0.00696
t half∗	10.954 ± 2.204	4.027 ± 0.266	6.927 ± 2.198	2.178	11.676	0.00765
t max#	1.826 ± 0.058	2.196 ± 0.084	−0.371 ± 0.078	−0.539	−0.202	0.00038
Vz∗	42.723 ± 2.147	62.171 ± 2.447	−19.448 ± 3.7	−27.441	−11.455	0.00016

∗ 2 × 2 cross-over design using *t*-test.

# 2 × 2 cross-over design using Mann-Whitney test.

**Table 6:** Comparison of pharmacokinetic parameters of withaferin A concentration in blood plasma with *Withania somnifera* extracts.

Parameters	WS-35	WS-2.5	LSM Difference ± SE	95% CI		P value
	LSM ± SE	LSM ± SE		Lower	Upper	
AUC 0-inf∗	748.947 ± 23.895	25.867 ± 0.541	723.08 ± 23.818	671.624	774.535	<0.001
AUC 0-t∗	587.476 ± 18.496	19.766 ± 0.42	567.71 ± 18.42	527.915	607.505	<0.001
AUC ext∗	161.471 ± 11.665	6.102 ± 0.401	155.369 ± 11.689	130.117	180.622	<0.001
Clearance∗	0.25 ± 0.008	7.197 ± 0.153	−6.947 ± 0.152	−7.275	−6.618	<0.001
Cmax∗	49.495 ± 1.244	5.869 ± 0.083	43.626 ± 1.259	40.906	46.346	<0.001
Ke∗	0.069 ± 0.003	0.25 ± 0.016	−0.181 ± 0.016	−0.216	−0.147	<0.001
Lambda Z∗	0.069 ± 0.003	0.25 ± 0.016	−0.181 ± 0.016	−0.216	−0.147	<0.001
Mean Residence Time∗	15.927 ± 0.532	4.353 ± 0.163	11.575 ± 0.567	10.349	12.8	<0.001
t half∗	10.348 ± 0.472	2.925 ± 0.185	7.423 ± 0.494	6.355	8.491	<0.001
t max#	2.277 ± 0.09	1.5 ± 0	0.777 ± 0.09	0.581	0.972	<0.001
Vz∗	3.713 ± 0.166	30.221 ± 1.832	−26.508 ± 1.826	−30.453	−22.564	<0.001

∗ 2 × 2 cross-over design using *t*-test.

# 2 × 2 cross-over design using Mann-Whitney test.

**Table 7:** Comparison of pharmacokinetic parameters of withanoside IV concentration in blood plasma with *Withania somnifera* extracts.

Parameters	WS-35	WS-2.5	LSM Difference ± SE	95% CI		P value
	LSM ± SE	LSM ± SE		Lower	Upper	
AUC 0-inf∗	92.516 ± 14.344	11.345 ± 0.354	81.171 ± 14.202	50.489	111.852	0.00007
AUC 0-t∗	36.985 ± 2.808	6.989 ± 0.12	29.996 ± 2.777	23.996	35.996	<0.001
AUC ext∗	55.531 ± 12.136	4.357 ± 0.362	51.174 ± 12.049	25.145	77.204	0.00095
Clearance∗	2.635 ± 0.353	16.529 ± 0.442	−13.894 ± 0.398	−14.753	−13.035	<0.001
Cmax∗	7.231 ± 0.416	2.668 ± 0.041	4.563 ± 0.405	3.689	5.437	<0.001
Ke∗	0.1 ± 0.013	0.302 ± 0.014	−0.202 ± 0.014	−0.232	−0.172	<0.001
Lambda Z∗	0.1 ± 0.013	0.302 ± 0.014	−0.202 ± 0.014	−0.232	−0.172	<0.001
Mean Residence Time∗	12.907 ± 1.621	4.178 ± 0.231	8.729 ± 1.574	5.328	12.13	0.00009
t half∗	8.856 ± 1.152	2.425 ± 0.181	6.431 ± 1.109	4.035	8.827	0.00006
t max#	1.763 ± 0.068	1.571 ± 0.066	0.192 ± 0.106	−0.037	0.421	0.09342
Vz∗	27.669 ± 2.626	56.376 ± 2.463	−28.707 ± 3.221	−35.666	−21.748	<0.001

∗ 2 × 2 cross-over design using *t*-test.

# 2 × 2 cross-over design using Mann-Whitney test.

**Table 8:** Comparison of pharmacokinetic parameters of total withanolides concentration in blood plasma with *Withania somnifera* extracts.

Parameters	WS-35	WS-2.5	LSM Difference ± SE	95% CI		P value
	LSM ± SE	LSM ± SE		Lower	Upper	
AUC 0-inf∗	815.036 ± 22.591	44.757 ± 0.573	770.278 ± 22.73	721.173	819.383	<0.001
AUC 0-t∗	662.568 ± 18.176	37.833 ± 0.504	624.735 ± 18.107	585.618	663.852	<0.001
AUC ext∗	152.468 ± 12.267	6.925 ± 0.162	145.543 ± 12.299	118.973	172.113	<0.001
Clearance∗	0.23 ± 0.007	4.144 ± 0.054	−3.914 ± 0.056	−4.035	−3.793	<0.001
Cmax∗	60.66 ± 1.086	10.789 ± 0.128	49.871 ± 1.128	47.433	52.308	<0.001
Ke∗	0.073 ± 0.003	0.369 ± 0.006	−0.296 ± 0.005	−0.307	−0.285	<0.001
Lambda Z∗	0.073 ± 0.003	0.369 ± 0.006	−0.296 ± 0.005	−0.307	−0.285	<0.001
Mean Residence Time∗	14.458 ± 0.565	3.503 ± 0.028	10.955 ± 0.559	9.747	12.162	<0.001
t half∗	9.756 ± 0.517	1.882 ± 0.03	7.874 ± 0.509	6.775	8.972	<0.001
t max#	2.277 ± 0.09	1.5 ± 0	0.777 ± 0.09	0.581	0.972	<0.001
Vz∗	3.207 ± 0.155	11.257 ± 0.244	−8.051 ± 0.204	−8.491	−7.611	<0.001

∗ 2 × 2 cross-over design using *t*-test.

# 2 × 2 cross-over design using Mann-Whitney test.

The AUC 0-t for withanolide A, withaferin A, withanoside IV and total withanolides for WS-35 were 2.68, 29.72, 5.29 and 17.51 fold higher than those for WS-2.5. The maximum concentration of withanolide A obtained at Tmax 1.826 from WS-35 was 1.62 fold higher than that obtained from WS-2.5. The mean residence time of withanolide A for WS-35 was 15.98 which was 2.56 times higher than that in WS-2.5. The maximum concentration of withaferin A obtained at Tmax 2.277 from WS-35 was 8.43 fold higher than that obtained from WS-2.5. The mean residence time of withaferin A for WS-35 was 15.927 which was 3.66 times higher than that in WS-2.5. The maximum concentration of withanoside IV obtained at Tmax 1.763 from WS-35 was 2.71 fold higher than that of WS-2.5. The mean residence time of withanoside IV for WS-35 was 12.907 which was 3.09 times higher than that of WS-2.5. The maximum concentration of total withanolides obtained at Tmax 2.277 from WS-35 was 5.62 fold higher than that from WS-2.5. The mean residence time of total withanolide A for WS-35 was 14.458 which was 4.13 times higher than that of WS-2.5. AUC 0-inf of withanolide A, withaferin A, withanoside IV and total withanolides for WS-35 respectively were 4.05, 28.95, 8.15 and 18.21 times higher than WS-2.5.

Comparing the area under curve of withanolide A, withaferin A, withanoside IV and total withanolides per 1 mg of WS-35 and WS-2.5, AUC 0-inf of WS-35 respectively would be 62.37, 446.37, 125.72 and 280.74 times better than WS-2.5 and AUC 0-t of withanolide A, withaferin A, withanoside IV and total withanolides for WS-35 respectively would be 41.36, 458.21, 81.58 and 269.99 times better than WS-2.5.

To establish bioequivalence, both AUC and C_max_ for the test product should be within 80%–120% of the reference product using a 90% confidence interval. 90% CI for AUC Ratios of WS-2.5, with respect to WS-35 do not fall in the range 80%–120%, indicating that WS-35 is not bioequivalent to WS-2.5. The average bioequivalence of the two products was found to be at a significance level of 0.05, and both confidence limits were outside the acceptance limits of 80 and 120. The Fieller’s confidence interval test and Schuirmann’s Wilcoxon-Mann-Whitney Equivalence test of pharmacokinetic parameters showed no statistically significant bioequivalence between WS-35 and WS-2.5 at α = 0.05 (Supplementary Tables S1–S2).

The investigational products were found to be safe and well tolerated by the administration of a single dose of ashwagandha extract in healthy adult male subjects under fasting conditions. There was no significant variability in the safety parameters (p > 0.05) during the treatment (Supplementary Table S3). No severe, serious, or life-threatening adverse events were reported during the course of the study.

## 4 Discussion

*Withania somnifera* of the Solanaceae family is the best-known plant that produces a group of secondary metabolites, known as withanolides. The general structure of withanolides is C28 ergostane with a side chain that forms a δ-lactone ring between C22 and C26. Based on their steroidal backbones, withanolides share an upstream biosynthetic pathway with phytosterols [13].

Bioavailability is defined as the fraction of the administered dose of a drug that reaches the systemic circulation [14]. In other words, bioavailability is the extent and rate at which the active ingredient or moiety in the product is absorbed and becomes available at the site of action. The extent and rate of drug absorption are usually measured using the area under the blood or plasma concentration-time curve (AUC) and maximum concentration (Cmax). The two products were considered pharmaceutical equivalents if they contained identical amounts of the same active ingredient. Two products are identified as pharmaceutical alternatives if they contain an identical therapeutic moiety, but not necessarily in the same amount or dosage form, or as the same salt or ester. Two products are said to be bioequivalent if they are pharmaceutical equivalents (i.e., similar dosage forms made, perhaps by different manufacturers) or pharmaceutical alternatives (i.e., different dosage forms). If their rates and extent of absorption do not show a significant difference, the active ingredient or active moiety in pharmaceutical equivalents or pharmaceutical alternatives become available at the site of action when administered at the same molar dose under similar conditions in an appropriately designed study. The products considered in this study are neither pharmaceutical equivalents nor alternatives. To claim bioequivalence in average bioavailability for the untransformed data, the ±20 rule requires that the ratio of the two true formulation averages be within (80%, 120%) limits. If the constructed confidence interval fell within the (80%, 120%) limits, then the two formulations were considered bioequivalent; otherwise, they were not bioequivalent. In our study, all the constructed 90% confidence intervals for the pharmacokinetic parameters of withanolide A, withaferin A, withanoside IV were outside the theoretical range, and both products were proved to be not bioequivalent.

Several factors affect the rate and extent of bioavailability following oral administration. In this study, ashwagandha extracts enriched with withanolide glycosides were compared with regular ashwagandha extracts standardized to contain equal amounts of total withanolides. Behl et al. (2020) analyzed the plasma concentration of Withaferin A mice and suggested the low bioavailability of withaferin A following oral administration of an aqueous solution of the crude extract [15,16]. The intestinal epithelium plays an important role in the absorption and transportation of medicinally active constituents of withanolides. Devkar et al. (2015) studied the intestinal permeability of withanolide constituents in *Withania somnifera* in an in vitro absorption model system using canine kidney cell culture, and reported the lowest permeability of Withaferin A whereas non-polar withanolides with low molecular weights, such as withanolide A, were found to be the most permeable [16,17].

Despite the standardized test products for total withanolide content, the pharmacokinetic profile varied significantly between the two products. One reason for the variability in the PK profile may be bioconversion. Withanolide glycosides undergo biotransformation and can be converted to withaferin A, which in turn increases the plasma concentration of withaferin A. The study results highlight the uniqueness of the composition of ashwagandha extract, as WS-35 is significantly richer in withanolide glycosides than WS-2.5. CYP450 enzymes in humans are involved in the metabolism of majority of drugs, with approximately 50% through CYP3A4 and 25% by CYP2D6 [18]. Therefore, it is possible that the drug accumulates to a toxic level due to the inhibition of CYP enzymes or rapid excretion due to the activation of CYP microsomal enzymes. Here, withanosides and withanolides acted as substrates for the CYP450 3A4 enzyme and non-substrates for CYP450 2C9 and CYP450 2D6 enzymes [19]. In the present study, withaferin A had the maximum oral bioavailability in systemic circulation. This may be due to the presence of withanolides, which regulate the hepatic metabolism of withaferin A and its biliary excretion.

Singh et al. [20] tested the bioavailability of withaferin A (purity 99%) by oral (25 mg/kg) and withanoside IV (2 mg/kg) routes in Sprague Dawley rats and found its oral bioavailability to be poor (approximately 5%) despite rapid distribution after i. v. administration. The distribution of a drug in the body is largely driven by its physicochemical properties, and partly by the contribution of transporter proteins [21]. The results of a phase I dose escalation study by Pires et al. [22] in 13 subjects did not detect withaferin A even at the highest dose tested. In this study, Withaferin A detected in the blood plasma of both ashwagandha extracts. One of the reasons for this difference could be attributed to the HPLC method by Pires et al. [22] which had a low specificity and sensitivity of 50 ng/ml, whereas our developed and validated LC-MS/MS method had an improved limit of quantification with an LLOQ of 1.0 ng/ml for withanoside IV and withanolide A and 0.5 ng/ml for withaferin A in plasma.

The area under the plasma level–time curve (AUC) is a measure of drug bioavailability. The AUC reflects the total amount of active drugs that reach systemic circulation. The AUC for total withanolides was the highest for WS-35, which was significantly higher than that for WS-2.5. The significantly high AUC for WS-35 was attributed to the high levels of maximum plasma concentration (C_max_) and longer T half of WS-35 compared to other test products. The significantly higher T-half of WS-35 compared to WS-2.5 indicates that WS-35, with its longer retention in the body, necessitates a lower dosage than WS-2.5. Such adjustment in dosage and are inevitable in optimizing the drug’s efficacy profile [23]. The Cmax and Tmax results indicated that withanosides, withaferin A, and withanolide A were rapidly absorbed by WS-35 compared to WS-2.5. The variability in the pharmacokinetic parameters may be attributed to differences in the concentration of withanolide glycosides between WS-35 and WS-2.5, as well as metabolic bioconversions, as withanolide glycosides undergo conversion into aglycones. The significant variation in T-max in the study reflect a complex interplay of several factors intrinsic to the study, such as individual sample characteristics, genetic diversities, and other unique variables specific to the samples under investigation. All of these factors collectively contribute to the observed variability in the time it takes for the drug to reach its peak concentration following drug administration. T-max can be influenced by a multitude of elements. Firstly, individual sample characteristics encompass a wide spectrum of variables, including but not limited to age, gender, weight, and underlying health conditions. These variables can affect how the body metabolizes and distributes the drug, ultimately influencing the time it takes to achieve peak concentration. Variations in genes responsible for drug metabolism and transport can lead to differences in the rate at which the drug is processed by the body, potentially affecting T-max [24].

A vast array of phytochemicals has been identified in ashwagandha. The Ashwgandha products available in the market were standardized using different analytical methods. USP-35-NF 30 [25] is generally accepted as a uniform analytical method but measures only withanoside IV, Physagulin D, 27-hydroxywithanone, withanoside V, withanoside VI, withaferin A, withastramonolide, withanolide A, withanone, and withanolide B in ashwagandha. Another validated and developed reverse-phase HPLC method provided precise differentiation and determination of withanolides and total withanolide glycosides present in the ashwagandha extract [26]. Such a robust and validated analytical method that measures various withanolides in ashwagandha would be helpful for standardizing ashwagandha extracts.

The toxicological assessment of WS-35 as conducted by Antony et al., in 2018 [27] has significantly contributed to establishing its safety profile. The acute oral toxicity study in rats unequivocally demonstrated the acute safety of WS-35 at dosages of up to 2000 mg/kg body weight, with an LD50 value exceeding 2000 mg/kg body weight, highlighting the remarkably high level of safety associated with WS-35. Furthermore, a 90-day repeated dose toxicity study in rats provided further evidence of the absence of toxic effects, as WS-35 was administered at a dosage level of 1000 mg/kg body weight. Notably, no observable toxic effects were discerned when compared to the control group, and both biochemical and hematological parameters remained within normal ranges throughout the study. Additionally, histopathological examinations of major organs revealed no abnormalities, reaffirming the safety of WS-35 for potential therapeutic applications.

### 4.1 Strength and limitations of the study

In this research study, a meticulously validated LC-MS-MS technique was utilized to precisely quantify the concentrations of three distinct withanolides: withaferin A, withanolide A, and withanoside IV in blood plasma. Remarkably, the concentrations of these compounds detected in the plasma samples fell well within the established validated range, underscoring a significant strength of this study. The lower limit of quantification (LLOQ) of withaferin A is 0.5 ng/ml, withanoside IV is 1 ng/ml and withanolide A is 1 ng/ml which shows that the sensitivity of the validated method is high.

One of the limitations of this study is that it focused solely on withanolides in plasma and did not account for their extensive in-vivo metabolism or the presence of secondary metabolites in urine or feces. Additionally, only three components: withanolide A, withanoside IV, and withaferin A was quantified in plasma samples. Furthermore, the study’s sample size was relatively small. Future research with a larger and more diverse sample size, encompassing a broader range of biological sexes and age groups, would enhance the generalizability of the findings and further strengthen the study conclusions.

## 5 Conclusion

Withanolides, an active class of bioactive compounds in *Withania somnifera* (Ashwagandha), were detected in human plasma, individually as withanoside IV, withaferin A, withanolide A, and total withanolides, after oral administration of WS extracts of different strengths, but normalized to the same dose of total withanolides. At the same dosage of total withanolides, the AUC 0-inf, Cmax, MRT, and t-half of WS-35 were 18.21, 5.62, 4.13 and 5.18 times greater than those of the WS-2.5 extract. This study indicated that the strength of the extract (as measured by the withanolide content) is critical for its oral bioavailability, as measured by the pharmacokinetic profile. The longer half-life and higher mean residence time of the higher strength extract WS-35, which contained 35% withanolide glycosides, demonstrated its enhanced oral bioavailability. The results of this study also highlight the lack of bioequivalence between the two WS extracts.

## Ethics approval and consent to participate

The study was conducted in accordance with the Declaration of Helsinki and approved by the Institutional Ethics Committee of Sai Sneh Hospital and Diagnostic Center, Pune. Before enrolling in the study, written informed consent was obtained from each participant by the principal investigator.

## Consent for publication

Not applicable.

## Data availability statement

Data will be made available on request.

## Funding

This research received no specific grant from any funding agency in the public, commercial, or not-for-profit sectors.

## CRediT authorship contribution statement

**Se-Kwon Kim:** Writing – review & editing, Writing – original draft, Validation, Formal analysis, Data curation. **Jayachandran Venkatesan:** Writing – review & editing, Writing – original draft, Validation, Formal analysis, Data curation. **Priyank Rathi:** Writing – original draft, Supervision, Project administration, Methodology, Investigation, Conceptualization. **Benny Antony:** Funding acquisition.

## Declaration of competing interest

The authors declare the following financial interests/personal relationships which may be considered as potential competing interests:Benny Antony has patent pending to Arjuna Natural Pvt Ltd. If there are other authors, they declare that they have no known competing financial interests or personal relationships that could have appeared to influence the work reported in this paper.

## List of Abbreviations

AUC: Area under curve
C_max_: Concentration maximum
HQC: Higher quality control
LLOQ: Lower limit of quantification
LQC: Lower quality control
MRT: Mean Residence Time
MQC: Middle quality control
PK: Pharmacokinetic
WS: *Withania somnifera*
