# Supplementary material for: Pharmacokinetics and bioequivalence of Withania somnifera (Ashwagandha) extracts – A double blind, crossover study in healthy adults
Source: Heliyon. 2023 Nov 28;9(12):e22843. doi: 10.1016/j.heliyon.2023.e22843 (PMC10746415; doi:10.1016/j.heliyon.2023.e22843)
Supplement: Multimedia component 1 [file mmc1.docx]

Supplementary Tables

| **Table S1. Equivalence tests of Withanolide A, Withaferin A, Withanoside IV and Total withanolides using 100(1 - 2α)% confidence intervals of the ratio using Fieller's C.I test** | | | | | | |
| --- | --- | --- | --- | --- | --- | --- |
| Parameter | Analyte | Lower Equivalence Limit | Lower 90% Confidence Limit | Upper 90% Confidence Limit | Upper Equivalence Limit | Conclude Equivalence at α = 0.050? |
| AUC 0-inf | Withanolide A | 80 | 380.096 | 428.947 | 120 | No |
|  | Withaferin A | 80 | 2820.07 | 2976.74 | 120 | No |
|  | Withanoside IV | 80 | 733.508 | 892.155 | 120 | No |
|  | Total withanolides | 80 | 1764.04 | 1880.79 | 120 | No |
| AUC 0-t | Withanolide A | 80 | 265.305 | 271.425 | 120 | No |
|  | Withaferin A | 80 | 2901.32 | 3049.15 | 120 | No |
|  | Withanoside IV | 80 | 510.245 | 547.952 | 120 | No |
|  | Total withanolides | 80 | 1726.53 | 1777.43 | 120 | No |
| AUC ext | Withanolide A | 80 | 622.562 | 648.914 | 120 | No |
|  | Withaferin A | 80 | 2357.62 | 3011.02 | 120 | No |
|  | Withanoside IV | 80 | 1175.67 | 1392.39 | 120 | No |
|  | Total withanolides | 80 | 2068.6 | 2347.39 | 120 | No |
| Clearance | Withanolide A | 80 | 28.689 | 41.052 | 120 | No |
|  | Withaferin A | 80 | 0 | 0 | 120 | No |
|  | Withanoside IV | 80 | 11.404 | 20.369 | 120 | No |
|  | Total withanolides | 80 | 3.231 | 7.856 | 120 | No |
| Cmax | Withanolide A | 80 | 152.141 | 172.125 | 120 | No |
|  | Withaferin A | 80 | 817.62 | 870.447 | 120 | No |
|  | Withanoside IV | 80 | 262.638 | 279.38 | 120 | No |
|  | Total withanolides | 80 | 546.372 | 578.781 | 120 | No |
| Ke | Withanolide A | 80 | 38.954 | 66.396 | 120 | No |
|  | Withaferin A | 80 | 15.869 | 39.819 | 120 | No |
|  | Withanoside IV | 80 | 25.549 | 40.435 | 120 | No |
|  | Total withanolides | 80 | 17.224 | 22.402 | 120 | No |
| Lambda Z | Withanolide A | 80 | 38.954 | 66.396 | 120 | No |
|  | Withaferin A | 80 | 15.869 | 39.819 | 120 | No |
|  | Withanoside IV | 80 | 25.549 | 40.435 | 120 | No |
|  | Total withanolides | 80 | 17.224 | 22.402 | 120 | No |
| Mean Residence Time | Withanolide A | 80 | 241.586 | 273.321 | 120 | No |
|  | Withaferin A | 80 | 340.905 | 394.351 | 120 | No |
|  | Withanoside IV | 80 | 299.491 | 320.669 | 120 | No |
|  | Total withanolides | 80 | 405.837 | 419.502 | 120 | No |
| t half | Withanolide A | 80 | 249.152 | 300.526 | 120 | No |
|  | Withaferin A | 80 | 318.477 | 397.564 | 120 | No |
|  | Withanoside IV | 80 | 367.781 | 368.534 | 120 | No |
|  | Total withanolides | 80 | 503.95 | 532.612 | 120 | No |
| Vz | Withanolide A | 80 | 59.725 | 78.636 | 120 | No |
|  | Withaferin A | 80 | 1.551 | 23.289 | 120 | No |
|  | Withanoside IV | 80 | 41.363 | 57.131 | 120 | No |
|  | Total withanolides | 80 | 24.877 | 32.07 | 120 | No |

| **Table S2. Schuirmann's Wilcoxon-Mann-Whitney Equivalence Test of Tmax using TOST (Two One-Sided Tests)** | | | | | | |
| --- | --- | --- | --- | --- | --- | --- |
| Analyte | Alternative Hypothesis | Lower Sum Ranks | Lower probability level | Upper Sum Ranks | Upper probability level | Conclude Equivalence at α = 0.050? |
| Withanolide A | -0.439 < Diff < 0.439 | 64 | 0.524 | 36 | 0.001 | No |
| Withaferin A | -0.300 < Diff < 0.300 | 92 | 0.001 | 90 | 0.999 | No |
| Withanoside IV | -0.314 < Diff < 0.314 | 89 | 0.002 | 51 | 0.068 | No |
| Total withanolides | -0.300 < Diff < 0.300 | 92 | 0.001 | 90 | 0.999 | No |

| **Table S3. Safety parameters** | | | | | | |
| --- | --- | --- | --- | --- | --- | --- |
|  | Screening | | Post-study | |  |  |
|  | Mean | SE | Mean | SE | Mean difference | p-value |
| Haemoglobin | 14.100 | 0.423 | 14.247 | 0.401 | 0.147 | 0.079 |
| RBC | 4.914 | 0.144 | 4.797 | 0.133 | -0.117 | 0.211 |
| PCV | 41.440 | 1.076 | 40.033 | 1.082 | -1.407 | 0.053 |
| WBC count | 7120.000 | 366.606 | 7106.667 | 361.408 | -13.333 | 0.869 |
| Neutrophils | 58.333 | 1.379 | 58.267 | 0.605 | -0.067 | 0.964 |
| Lymphocytes | 35.667 | 1.256 | 35.400 | 0.638 | -0.267 | 0.855 |
| Eosinophils | 2.200 | 0.107 | 2.333 | 0.126 | 0.133 | 0.433 |
| Monocytes | 3.800 | 0.243 | 4.000 | 0.218 | 0.200 | 0.424 |
| Platelet count | 279666.667 | 12272.178 | 262666.667 | 17816.703 | -17000.000 | 0.346 |
| SGOT | 29.627 | 1.936 | 29.600 | 1.931 | -0.027 | 0.104 |
| SGPT | 30.400 | 1.716 | 30.353 | 1.730 | -0.047 | 0.068 |
| Alkaline phosphatase | 68.600 | 4.573 | 68.067 | 4.664 | -0.533 | 0.342 |
| total bilurubin | 0.836 | 0.075 | 7.696 | 6.951 | 6.860 | 0.339 |
| Blood urea | 21.140 | 1.529 | 21.107 | 1.524 | -0.033 | 0.136 |
| Creatinine | 0.889 | 0.022 | 0.887 | 0.022 | -0.002 | 0.082 |
